# Supplementary material for: Opposite regulation of inhibition by adult-born granule cells during implicit versus explicit olfactory learning
Source: eLife. 2018 Feb 28;7:e34976. doi: 10.7554/eLife.34976 (PMC5829916; doi:10.7554/eLife.34976)
Supplement: Transparent reporting form [file elife-34976-transrepform.docx]

***eLife’s* transparent reporting form**

We encourage authors to provide detailed information *within their submission* to facilitate the interpretation and replication of experiments. If you have any questions, please contact us: [editorial@elifesciences.org](mailto:editorial@elifesciences.org).

**Sample-size estimation**

- You should state whether an appropriate sample size was computed when the study was being designed
- You should state the statistical method of sample size computation and any required assumptions
- If no explicit power analysis was used, you should describe how you decided what sample (replicate) size (number) to use

Please outline where this information can be found within the submission (e.g., page numbers or figure legends), or explain why this information doesn’t apply to your submission:

•No statistical methods were used to predetermine sample sizes, but our sample sizes were similar to those reported in previous publications (Alonso et al 2012; Mandairon et al 2011; Moreno et al 2012; Gschwend et al 2015; Daroles et al 2016).

• The sample size is stated in each legend figure.

**Replicates**

- You should report how often each experiment was performed
- You should include a definition of biological versus technical replication
- The data obtained should be provided and sufficient information should be provided to indicate the number of independent biological and/or technical replicates
- If you encountered any outliers, you should describe how these were handled
- Criteria for exclusion/inclusion of data should be clearly stated
- High-throughput sequence data should be uploaded before submission, with a private link for reviewers provided (these are available from both GEO and ArrayExpress)

Please outline where this information can be found within the submission (e.g., page numbers or figure legends), or explain why this information doesn’t apply to your submission:

• Learning paradigms and quantification of neurogenesis performed in the study are biological replicates of previous published data (Moreno et al 2009; Sultan et al 2010; Mandairon et al 2011; Moreno et al 2012). Morphological analysis after implicit learning biologically replicates Daroles et al 2016). Other experiments are original.

By biological replicate, we mean that different group of animals were submitted to the same protocol. Technical replicates were performed for some groups for which BrdU positive cell density and dendritic spine density were analyzed twice.

• No outlier have been considered.

• In the behavioral experiments, no data could be collected for 3 trials out of 125 in the enriched group and the corresponding animals (3) were removed from the statistical analysis. No other exclusion criteria were used in any of the experiments.

**Statistical reporting**

- Statistical analysis methods should be described and justified
- Raw data should be presented in figures whenever informative to do so (typically when N per group is less than 10)
- For each experiment, you should identify the statistical tests used, exact values of N, definitions of center, methods of multiple test correction, and dispersion and precision measures (e.g., mean, median, SD, SEM, confidence intervals; and, for the major substantive results, a measure of effect size (e.g., Pearson's r, Cohen's d)
- Report exact p-values wherever possible alongside the summary statistics and 95% confidence intervals. These should be reported for all key questions and not only when the p-value is less than 0.05.

• All analyses were performed using Statistica. A Kolmogorov-Smirnov test was used to assess normality of the data. Non-parametric tests (Mann-Whitney Friedman ANOVA or Wilcoxon tests for paired data) were used for independent samples and parametric tests (t-test for paired comparisons) for samples with normal distribution. For all parametric test, homogeneity of variance was tested using Levene’s test.

Data collection and animal assignation to the various experimental groups were randomized.

• Raw data are presented in the figures in dot-plot format (individual data points displayed along with central and dispersion statistics)

• For each figure, the statistical test used, the exact value of N and the SEM are given in the legend.

• The exact p value is always given in the legend of the figure.

Please outline where this information can be found within the submission (e.g., page numbers or figure legends), or explain why this information doesn’t apply to your submission:

(For large datasets, or papers with a very large number of statistical tests, you may upload a single table file with tests, Ns, etc., with reference to page numbers in the manuscript.)

**Additional data files (“source data”)**

- We encourage you to upload relevant additional data files, such as numerical data that are represented as a graph in a figure, or as a summary table
- Where provided, these should be in the most useful format, and they can be uploaded as “Source data” files linked to a main figure or table
- Include model definition files including the full list of parameters used
- Include code used for data analysis (e.g., R, MatLab)
- Avoid stating that data files are “available upon request”

Please indicate the figures or tables for which source data files have been provided:

• A data file has been uploaded as Source data
